# A Basic Guide to Real Time PCR in Microbial Diagnostics: Definitions, Parameters, and Everything

**DOI:** 10.3389/fmicb.2017.00108

**Published:** 2017-02-02

**Authors:** Petr Kralik, Matteo Ricchi

**Affiliations:** ^1^Department of Food and Feed Safety, Veterinary Research InstituteBrno, Czechia; ^2^Istituto Zooprofilattico Sperimentale della Lombardia e dell'Emilia Romagna “Bruno Ubertini,” National Reference Centre for ParatuberculosisPiacenza, Italy

**Keywords:** quantitative PCR, limit of detection, limit of quantification, accuracy, precision, trueness

## Abstract

Real time PCR (quantitative PCR, qPCR) is now a well-established method for the detection, quantification, and typing of different microbial agents in the areas of clinical and veterinary diagnostics and food safety. Although the concept of PCR is relatively simple, there are specific issues in qPCR that developers and users of this technology must bear in mind. These include the use of correct terminology and definitions, understanding of the principle of PCR, difficulties with interpretation and presentation of data, the limitations of qPCR in different areas of microbial diagnostics and parameters important for the description of qPCR performance. It is not our intention in this review to describe every single aspect of qPCR design, optimization, and validation; however, it is our hope that this basic guide will help to orient beginners and users of qPCR in the use of this powerful technique.

## Introduction

The phrase “Polymerase chain reaction” (PCR) was first used more than 30 years ago in a paper describing a novel enzymatic amplification of DNA (Saiki et al., 1985). The first applications of PCR were rather unpractical due to the usage of thermolabile Klenow fragment for amplification, which needed to be added to the reaction after each denaturation step. The crucial innovation which enabled routine usage of PCR was utilization of thermostable polymerase from *Thermus aquaticus* (Saiki et al., 1988). This improvement, together with the availability of PCR cyclers and chemical components, led to the worldwide recognition of PCR as the tool of choice for the specific enzymatic amplification of DNA *in vitro*. It must be noted that the general concept of PCR, which includes primers, DNA polymerase, nucleotides, specific ions, and DNA template, and consisting of cycles that comprise steps of DNA denaturation, primer annealing, and extension, have not been changed since 1985. The invention of PCR has greatly boosted research in various areas of biology and this technology has significantly contributed to the current level of human knowledge in many spheres of research.

The most substantial milestone in PCR utilization was the introduction of the concept of monitoring DNA amplification in real time through monitoring of fluorescence (Holland et al., 1991; Higuchi et al., 1992). In real time PCR (also denoted as quantitative PCR—qPCR; usage of RT-PCR is inappropriate as this abbreviation is dedicated to reverse transcription PCR), fluorescence is measured after each cycle and the intensity of the fluorescent signal reflects the momentary amount of DNA amplicons in the sample at that specific time. In initial cycles the fluorescence is too low to be distinguishable from the background. However, the point at which the fluorescence intensity increases above the detectable level corresponds proportionally to the initial number of template DNA molecules in the sample. This point is called the quantification cycle (C_q_; different manufactures of qPCR instruments use their own terminology, but since 2009, the term C_q_ is used exclusively) and allows determination of the absolute quantity of target DNA in the sample according to a calibration curve constructed of serially diluted standard samples (usually decimal dilutions) with known concentrations or copy numbers (Yang and Rothman, 2004; Kubista et al., 2006; Bustin et al., 2009).

Moreover, qPCR can also provide semi-quantitative results without standards but with controls used as a reference material. It this case, the observed results can be expressed as higher or lower multiples with reference to control. This application of qPCR has been extensively used for gene expressions studies (Bustin et al., 2009), but did not obtain the same success in microbiology quantification since it is unable to produce absolute quantitative values.

There are two strategies for the real time visualization of amplified DNA fragments—non-specific fluorescent DNA dyes and fluorescently labeled oligonucleotide probes. These two approaches were developed in parallel (Holland et al., 1991; Higuchi et al., 1992) and are used in pathogen detection; however, probe-based chemistry prevails. This is due to its higher specificity mediated by the additional oligonucleotide—the probe—and the lower susceptibility to visualize non-specific PCR products, e.g., primer dimers (Bustin, 2000; Kubista et al., 2006).

To fully understand the possibilities of qPCR in detecting and quantifying target DNA in samples it is essential to describe the mathematical principle of this method. The PCR is an exponential process where the number of DNA molecules theoretically doubles after each cycle (if the efficiency of the reaction is 100%). More generally, the amplification reaction follows this equation:

(1) $$\begin{array}{l} N_{n}\;=\;N_{0}\;\times \;{\left(1\;+\;E\right)}^{n} \end{array}$$

where N_n_ is the number of PCR amplicons after n cycles, N_0_ is the initial number of template copies in the sample, E is the PCR efficiency that can assume values in the range from 0 to 1 (0–100%) and n is number of cycles. In a scenario where there is initially one copy of the template in the reaction and PCR efficiency is 100%, it is possible to simplify the equation as follows:

(2) $$\begin{array}{l} N_{n}\;=\;2^{n} \end{array}$$

If a calibration curve is run, usually 10-fold serial dilutions are used. The difference in C_q_ values between two 10-fold serial dilutions could be expressed as

(3) $$\begin{array}{l} 10=\;2^{n} \end{array}$$

Then *n* = 3.322. When E should be determined the (1) is starting point and the equation is

(4) $$\begin{array}{l} E\;=\;10^{-\left(\frac{1}{n}\right)}\;-\;1 \end{array}$$

If n is taken to be 3.322, then *E* = 1, i.e., 100%.

**Figure 1:**
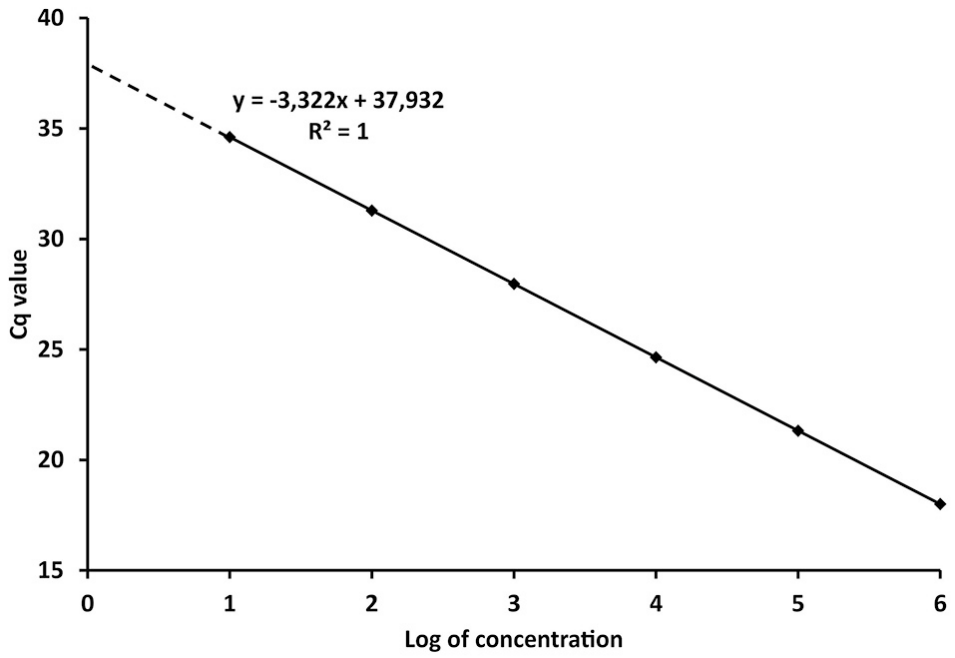
**Model calibration curve with the regression equation (characterized by the slope and intercept) and regression coefficient**.

The PCR efficiency is therefore a significant factor for the quantification of the target DNA in unknown samples. The reliability of the calibration curve in enabling quantification is then determined by the spacing of the serial dilutions. If the Log_10_ of the concentration or copy number of each standard is plotted against its C_q_ value (Figure 1), the E can be derived from the regression equation describing the linear function:

(5) $$\begin{array}{l} y\;=\;kx\;+\;c \end{array}$$

Where x and y, the concentration/amount of target and C_q_ values respectively, characterize the coordinates in the plot, k is the regression coefficient or slope and c is the intercept. Taking the model regression equation from Figure 1, the slope is −3.322, which mean that *E* = 100% according to (4). The intercept shows the C_q_ value when one copy would be theoretically detected (Kubista et al., 2006; Johnson et al., 2013). The concentration or amount of target nucleic acid in unknown samples is then calculated according to the C_q_ value through Equation (5).

From the definitions above it is evident that C_q_ values are instrumental readings, and must be recalculated to values with specific units, e.g., copies of organism, ng of DNA, various concentrations, etc., (Bustin et al., 2009; Johnson et al., 2013). However, referral to C_q_ values in scientific papers is widespread and interpretations based on C_q_ values can lead to misleading conclusions. Concentrations in qPCR are expressed in the logarithmic scale (Figure 1) and C_q_ differences between 10-fold serial dilutions are theoretically always 3.322 cycles. Therefore, although the numerical difference between C_q_ 20 and 35 is rather negligible, the difference in real numbers (copies, ng) is almost five orders of magnitude (Log_10_).

This feature must be reflected in the subsequent calculations. For example, the coefficient of variation (CV, ratio between standard deviation and mean) calculated from the C_q_ values and real numbers results in profoundly different results. The same applies for any statistical tests where C_q_ values are used, even for cases where the logarithm of C_q_ values is used for the normalization of data before the statistical evaluation. The correct procedure should include initial recalculation to real numbers followed by logarithmic transformation.

## Pros and cons of using qPCR in detection and quantification of pathogens

Since PCR is capable of amplifying a specific fragment of DNA, it has been used in pathogen diagnostics. With the increasing amount of sequencing data available, it is literally possible to design qPCR assays for every microorganism (groups and subgroups of microorganisms, etc.) of interest. The main advantages of qPCR are that it provides fast and high-throughput detection and quantification of target DNA sequences in different matrices. The lower time of amplification is facilitated by the simultaneous amplification and visualization of newly formed DNA amplicons. Moreover, qPCR is safer in terms of avoiding cross contaminations because no further manipulation with samples is required after the amplification. Other advantages of qPCR include a wide dynamic range for quantification (7–8 Log_10_) and the multiplexing of amplification of several targets into a single reaction (Klein, 2002). The multiplexing option is essential for detection and quantification in diagnostic qPCR assays that rely on the inclusion of internal amplification controls (Yang and Rothman, 2004; Kubista et al., 2006; Bustin et al., 2009).

qPCR assays are used not only for the detection, but also to determine the presence of specific genes and alleles, e.g., typing of strains and isolates, antimicrobial resistance profiling, toxin production, etc., However, the mere presence of genes responsible for resistance to antimicrobial compounds or fungal toxin production does not automatically mean their expression or production. Therefore, although qPCR-based typing tests are faster, their results should be correlated with phenotypic and biochemical tests (Levin, 2012; Osei Sekyere et al., 2015).

As for the microbial diagnostics, there are different considerations in detecting and quantifying viral, bacterial, and parasitic agents. These considerations are based on the target (DNA or RNA), cultivability, interpretation of results, and clinical significance of qPCR results.

qPCR plays an important role in the detection, quantification, and typing of viral pathogens. This is because detection of important clinical and veterinary viruses using culture methods is time-consuming or impossible, while ELISA tests are not universally available and suffer from comparatively low sensitivity and specificity. qPCR (with the inclusion of reverse transcription for the diagnostics of RNA viruses) provides the appropriate sensitivity and specificity (Hoffmann et al., 2009). Moreover, determination of the viral load by (RT)-qPCR is used as an indicator of the response to antiviral therapies (Watzinger et al., 2006). For these reasons (RT)-qPCR has become an indispensable tool in virus diagnostics (Yang and Rothman, 2004).

The situation is similar in the case of intestinal protozoan diagnostics (Rijsman et al., 2016). The gold standard technique for the detection of protozoan agents, the microscopic examination of feces, is laborious, time-consuming, and requires specifically trained personnel. Similarly, ELISA testing suffers from low sensitivity and specificity (Rijsman et al., 2016). Therefore, qPCR is now emerging as a powerful tool in the routine detection, quantification, and typing of intestinal parasitic protozoa.

In contrast to viral and protozoan detection and quantification, many bacteria of clinical, veterinary, and food safety significance, can be cultured. For this reason, culture is considered as the gold standard in bacterial detection and quantification. However, in cases when critical and timely intervention for infectious disease is required, the traditional, slow, and multistep culture techniques cannot provide results in a reasonable time. This limitation is compounded by the necessity of culturing fastidious pathogens and additional testing (species determination, identification of virulence factors, and antimicrobial resistance). qPCR is capable of providing the required information in a short time; however, the phenotypic and biochemical features must be confirmed from bacterial isolates (Yang and Rothman, 2004).

In food safety, all international standards for food quality rely on the determination of pathogenic microorganisms using traditional culture methods. qPCR techniques represent an excellent alternative to existing standard culture methods as they enable reliable detection and quantification (for several pathogens) and harbor many other advantages as discussed above. However, there are limitations with respect to the sensitivity of assays based on qPCR. As culture methods rely on the multiplication of bacteria during the pre-culture steps (pre-enrichment), samples for DNA isolation usually initially contain very low numbers of target bacteria (Rodriguez-Lazaro et al., 2013). This limitation leads to the most important disadvantage of qPCR, which is its inherent incapability of distinguishing between live and dead cells. The usage of qPCR itself is therefore limited to the typing of bacterial strains, identification of antimicrobial resistance, detection, and possibly quantification in non-processed and raw food. It is important to note that processed food can still contain amplifiable DNA even if all the potentially pathogenic bacteria in food are devitalized and the foodstuff is microbiologically safe for consumption (Rodriguez-Lazaro et al., 2013). To overcome this problem, a pre-enrichment of sample in culture media could be placed prior to the qPCR. This step may include non-selective enrichment in buffered peptone water or specific selective media for the respective bacterium. This procedure is primarily intended to allow resuscitation/recovery and subsequent multiplication of the bacteria for the downstream qPCR detection; the second advantage is dilution and elimination of possible PCR inhibitors present into the sample (presence of salts, conservation substances, etc.). The extraction of the DNA from the culture media is easier than that from the food samples, which are much more heterogeneous in terms of composition (Margot et al., 2015).

Although qPCR itself cannot distinguish among viable and dead cells attempts have been made to adapt qPCR for viability detection. It was shown that RNA has low stability and should be degraded in dead cells within minutes. However, the correlation of cell viability with the persistence of nucleic acid species must be well characterized for a particular situation before an appropriate amplification-based analytical method can be adopted as a surrogate for more traditional culture techniques (Birch et al., 2001). Moreover, difficulties connected with RNA isolation from samples like food, feces or environmental samples can provide false-negative results especially when low numbers of target cells are expected.

Another option for determination of viability using qPCR is the deployment of intercalating fluorescent dyes like propidium monoazide (PMA) and ethidium monoazide (EMA; Nocker and Camper, 2009). In these methods, the criterion for viability determination is membrane integrity. Metabolically active cells (regardless of their cultivability) with full membrane integrity keep the dyes outside the cells and are therefore considered as viable. However, if plasma membrane integrity is compromised, the dyes penetrate the cells, or react with the DNA outside of dead cells. The labeled DNA is then not available for the amplification by qPCR and the difference between treated and untreated cells provides information about the proportion of viable cells in the sample. The limitation of this method is the necessity to have the cells in a light-transparent matrix, e.g., water samples, cell cultures, etc., as the intercalation of the dye to DNA requires exposure to light. Therefore, samples of insufficient light transparency do not permit the application of these dyes. There is a preference for PMA over EMA, as it was shown that EMA penetrates the membranes of live bacterial cells (Nocker et al., 2006).

Moreover, another topic we want to just to mention here is the generation and use of standards required for the calibration curves. In general, two are the most diffused approaches for the generation of calibration curves. One employs dilutions of target genomic nucleic acid and the other plasmid standards. Both strategies can lead to a final quantification of the target, but plasmids containing specific target sequences offer the advantages of easy production, stability, and cheapness. On the other hand, in principle, PCR efficiency obtained by plasmid standards sometimes could differ compared to the efficiency obtained using genomic standard, which instead, for organisms fastidious to growth, could be isolated only starting from a given matrix, and thus susceptible to degradation and losses (Chaouachi et al., 2013). Finally, the production and validation of international quantification standards for qPCR assays is technically demanding and these standards are currently available only for a few targets (Pavšič et al., 2015).

## qPCR parameters in microbial detection and quantification

### Analytical specificity (selectivity)

This parameter in qPCR refers to the specificity of primers for target of interest. Analytical specificity consists of two concepts: inclusivity describes the ability of the method to detect a wide range of targets with defined relatedness e.g., taxonomic, immunological, genetic composition (Anonymous, 2009, 2015a). Another definition describes inclusivity as the strains or isolates of the target analyte(s) that the method can detect (Anonymous, 2012). ISO 16140 and other standards recommend that inclusivity should be determined on 20–50 well-defined (certified) strains of the target organism (Anonymous, 2009, 2011, 2012, 2015a; Broeders et al., 2014), or for *Salmonella*, it is recommended that 100 serovars should be included for inclusivity testing (Anonymous, 2012).

On the other hand, exclusivity describes the ability of the method to distinguish the target from similar but genetically distinct non-targets. In other words, exclusivity can also be defined as the lack of interference from a relevant range of non-target strains, which are potentially cross-reactive (Anonymous, 2009, 2011, 2012, 2015a). The desirable number of positive samples in exclusivity testing is zero (Johnson et al., 2013).

### Analytical sensitivity (limit of detection, LOD)

Many official documents have discussed theories and procedures for the correct definition of the LOD for different methods. A general consensus was reached around the definition of the LOD as the lowest amount of analyte, which can be detected with more than a stated percentage of confidence, but, not necessarily quantified as an exact value (Anonymous, 2011, 2013, 2014). In this regard, the confidence level obtained or requested for the definition of LOD can reflect the number of replicates (both technical and experimental) needed by the assay in order to reach the requested level of confidence (e.g., 95%). It is clear that the more replicates are tested, the narrower will be the interval of confidence. Another definition describes the LOD as the lowest concentration level that can be determined as statistically different from a blank at a specified level of confidence. This value should be determined from the analysis of sample blanks and samples at levels near the expected LOD (Anonymous, 2015a). However, it should be noted that LOD definitions described above were reported for chemical methods, and are not perfectly suited for PCR methods (Burns and Valdivia, 2008). This is because, for limited concentrations of analyte (nucleic acids), the output of the reaction can be a success (amplification), or a failure (no amplification at all), without any blank, or critical level at which it is possible to set a cut-off value over which the sample can be considered as positive one. Moreover, it should be remembered here that, by definition, a blank sample should never be positive in PCR.

**Figure 2:**
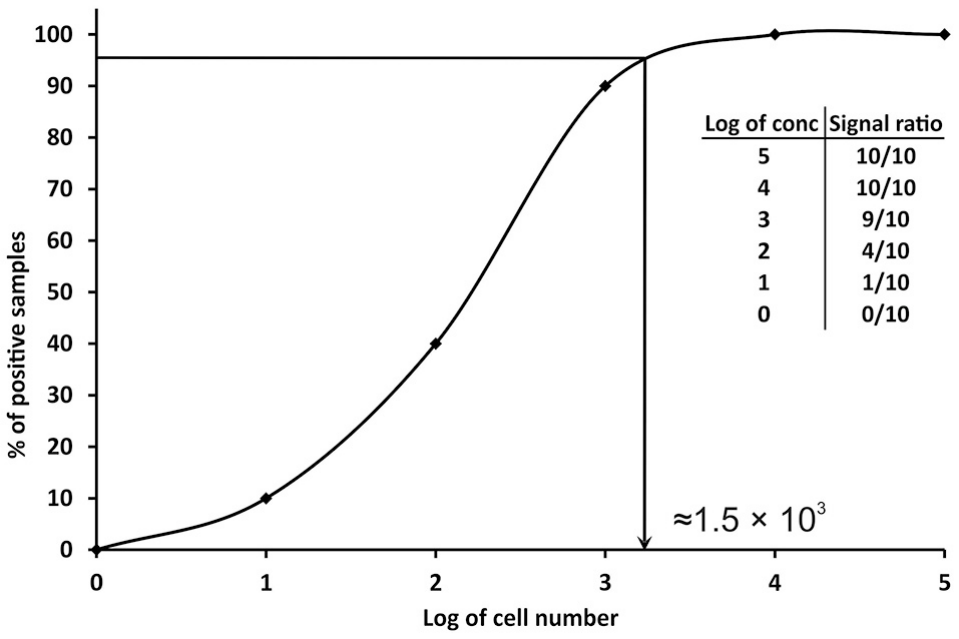
**Graphical representation of the determination of limit of detection (LOD) in qPCR**. The data in the table show the number of positive samples/all analyzed samples (signal ratio).

Since the definitions reported above are not practicable for PCRs, other approaches have been proposed. A conservative approach is to consider the LOD value as the minimum concentration of nucleic acid or number of cells, which always gives a positive PCR result in all replicates tested, or in the major part (over 95%) of them (Nutz et al., 2011). In practice, multiple aliquots of a specific matrix are spiked with serial dilutions of the target organism and undergo the whole process of nucleic acid isolation and qPCR. The LOD is then defined as the spike amount of target organism in dilution that could be detected in 95% of replicates. For example, 10 replicates of milk samples were spiked with serial dilutions of *Campylobacter jejuni* in amounts of 10^5^–10^0^ cells per 1 ml of milk. The experimentally determined LOD of the method for the detection of *C. jejuni* in milk is approximately 1.56 × 10^3^ cells/ml of milk (Figure 2). In order to better define the most precise value, more dilutions can be tested before reaching a final LOD value as close as possible to the real one. The number of replicates tested should be at least six (Slana et al., 2008; Kralik et al., 2011); however, the more replicates (10 or 15 and more; Ricchi et al., 2016) performed, the higher level of confidence of the LOD that can be achieved (Anonymous, 2015b).

According to the Poisson distribution, it was concluded that the LOD for PCR cannot be lower than at least three copies of the nucleic acid targets (Bustin et al., 2009; Johnson et al., 2013). However, this value refers to the theoretical LOD of the qPCR methodology, which is capable of detecting a single target DNA molecule in the sample. Assuming this, such LOD for all optimized qPCR assays will be similar. Therefore, as stated above, the LOD must be related to the whole method that includes nucleic acid preparation and qPCR. Only under these conditions can it represent a valid parameter that describes the features of the respective qPCR method (Anonymous, 2015a).

However, sometimes it is not possible to obtain large numbers of replicates, for both financial and technical reasons. To overcome these problems, an increasing number of reports utilize Probit or Logit approaches for determining the LOD for PCR methods (Burns and Valdivia, 2008; Anonymous, 2014; Pavšic et al., 2016; Ricchi et al., 2016). Briefly, both mathematical functions are regressions used to analyse binomial response variables (positive or negative) and are able to transform the sigmoid dose-response curve, typical of a binomial variable, to a straight line that can then be analyzed by regression either through least squares or maximum likelihood methods. The final end-point of the analysis is a concentration (coupled with relative intervals of confidence), associated to a probability (e.g., 95%) to detect the nucleic acid. Moreover, Probit regression is exploitable only for normally distributed data, while Logit function can also be used for data not normally distributed; however, in this context, both functions have the same meaning.

Finally, it must be noted that LOD is not a limiting value and therefore, that C_q_ values below the LOD cannot automatically be considered as negative. From the definition of LOD, it is evident that values below the LOD are absolutely valid in terms of microorganism presence; however, the probability of their repeated detection is lower than 95%. This feature is connected with the Poisson distribution when working with small numbers.

### Limit of quantification (LOQ)

The documents already cited for the LOD definitions also contain analog definitions for the LOQ. The LOQ was defined as the smallest amount of analyte, which can be measured and quantified with defined precision and accuracy under the experimental conditions by the method under validation (Armbruster and Pry, 2008; Anonymous, 2011, 2013). An alternative definition is that the LOQ is the lowest amount or concentration of analyte that can be quantitatively determined with an acceptable level of uncertainty (Anonymous, 2015a). It is clear that, according to the previous definitions, the LOQ can never be lower than the LOD.

In practice, the LOQ is determined as is the LOD, on replicates of spiked samples, but the assessment of results is quantitative. Numerically, the LOQ is defined as the lowest concentration of analyte, which gives a predefined variability, generally reported as the coefficient of variation (CV). For qPCR, this value has been proposed to be fixed under 25% (Broeders et al., 2014; Dreo et al., 2014; Anonymous, 2015b; Pavšic et al., 2016), bearing in mind that the C_q_ values must be recalculated to copies or g of nucleic acids before performing the evaluation of the CV (Bustin et al., 2009; Johnson et al., 2013). Hoverer, this value was proposed based on the experience accrued in GMO detection laboratories (Broeders et al., 2014; Anonymous, 2015b), and there is no general agreement regarding any technical standards for molecular methods in microbiology. Therefore, we propose here to define the LOQ in the molecular diagnosis of microorganisms as the lowest concentration, amount, or number of analytes with a *CV* < 25%.

Another approach for the determination of the LOQ of qPCR is based on the use of the Youden index (J) and receiver operating characteristic curves (Nutz et al., 2011). This last index was defined as *J* = sensitivity + specificity −1 (Fluss et al., 2005). A series of spiked samples with different concentrations of target DNA were analyzed and the *J*-values were calculated for each PCR cycle. The LOQ was then fixed as the concentrations of DNA where the *J*-values were highest (Nutz et al., 2011).

Finally, an issue that should be addressed for the determination of the LOQ as well as LOD is the efficiency of recovery of target molecules during the nucleic acid extraction phases. Generally, nucleic acids are extracted from different complex matrices, like food, feces, or other samples using different procedures. The efficiency of DNA recovery is usually around 30% and lower (Slana et al., 2008; Kralik et al., 2011; Ricchi et al., 2016) and neglecting this parameter leads to underestimation of the true number of target microorganisms in the original sample, which is then reflected by the lower LOD and LOQ values. Therefore, determination of DNA isolation efficiency should be part of the LOD and LOQ. DNA isolation efficiency is a quotient between the number of microorganisms recovered after the entire procedure (nucleic acid extraction + qPCR) and the number of microorganisms used for spiking the negative matrices (Slana et al., 2008; Kralik et al., 2011; Ricchi et al., 2016). Due to the fact that these data are provided during the determination of the LOD and LOQ, it is not necessary to perform additional experiments. It is recommended that the median of mean DNA isolation values from different dilutions is used as the practical overall DNA isolation efficiency (Kralik et al., 2011).

Similarly to the LOD, quantity can also be assessed in samples with numbers of organisms or concentrations of DNA lower than the LOQ, but the confidence of such quantification will be lower than that declared by the definition of LOQ. Moreover, there are possibilities of how to refer to such quantities in terms of semi-quantitative interpretation, e.g., range of values (10^2^–10^1^ cells/g).

### Amplification efficiency of qPCR (E)

This parameter was mentioned above in the section dedicated to the mathematical description of qPCR (Equation 4). PCR efficiency should be in the range of 0–1 (0–100%); when *E* = 1 this means that the number of newly formed DNA amplicons is doubled in each cycle. This is difficult to reach repeatedly over time. In practice, this parameter is likely to be in the range 90–105% (Johnson et al., 2013). This parameter can be estimated from the slope of the calibration curve.

In connection to this issue, the lowest and highest concentrations of the standard included in the calibration curve, which can be truly quantified, should be determined according to the linear dynamic range of over at least 6 Log_10_. The dynamic range is defined by the MIQE guidelines as the range over which a reaction is linear (Bustin et al., 2009).

The determination of PCR efficiency by the standard curve actually provides two pieces of information. If an inhibitor would be present in the most concentrated sample, there would be a visible increase in C_q_ values in these and therefore a diminishment of the 3.322 C_q_ span at higher concentrations. However, this is not a frequent phenomenon, as standards are usually well-characterized and therefore, any inhibition is rather unlikely. If there would be a similar situation in lower concentration samples, this suggests a possible pipetting error rather than the presence of inhibitors. An important function to assess this is the coefficient of determination (*R*^2^ value), that should be higher than 0.98 (Johnson et al., 2013). In reality, it is much more important to determine the PCR inhibition and subsequent diminishment of the PCR efficiency in analyzed samples. There are approaches based on the analysis of the fluorescent curve of each sample by specific software (LinRegPCR), which can calculate the PCR efficiency of each sample without the series of dilutions. However, this approach is not flawless as it does not take into account all possible variables that can affect the analysis (Ruijter et al., 2009).

### Accuracy of PCR

The following parameters of qPCR deal with ways of how to compare novel qPCR methods with reference methods or materials. Accuracy is defined as a measure of the degree of conformity of a value generated by a specific procedure to the assumed or accepted true value (Anonymous, 2015a). In other words, accuracy describes the level of agreement between reference and measured values. There are several aspects that need to be considered in terms of defining accuracy. In binary classification tests (qualitative detection), the samples analyzed by a novel (alternative) test that needs to be verified (typically a novel qPCR assay) are categorized according to their concordance with the reference method in four basic categories (Table 1). This division originates from the statistical classification known as error matrix and allows determination of several parameters that describe the diagnostic potential of the qPCR method.

**Table 1:** **Parameters for comparison of qPCR results with a reference method in a 2 × 2 error matrix contingency table**.

		**Reference method**	
		**Positive**	**Negative**
Alternative method	Positive	TP	FP
	Negative	FN	TN

*TP—True positive—Positive sample correctly identified as positive*.

*TN—True negative—Negative sample correctly identified as negative*.

*FP—False positive—Negative sample wrongly identified as positive*.

*FN—False negative—Positive sample wrongly identified as negative*.

Diagnostic sensitivity, which is described as TP/(TP + FN), refers to the ability of the new test to correctly identify samples identified by the reference method as positive. The lower the diagnostic sensitivity, the poorer will be the inclusivity of the tested qPCR. Another explanation could be that the analytical sensitivity (LOD) of the reference method is higher than the tested qPCR.

Diagnostic specificity is defined as the TN/(TN + FP) and refers to the ability of the test to correctly identify samples that were found to be negative by the reference method. The lower the diagnostic specificity, the poorer will be the exclusivity of the tested qPCR. Another explanation could be that the sensitivity of the reference method is quite bad, and the new qPCR method is capable of identifying more positive samples than the reference method.

Relative accuracy is defined as the (TP + TN)/(TP + TN + FP + FN) and describes the proportion of all correctly identified samples among all samples (Anonymous, 2009). If no FN and FP are detected, then it is 100%. In all other cases, this value is lower than 100%.

In quantitative determination, the accuracy numerically describes the distance of the value from the novel tested qPCR and some reference (true) value. For this reason, accuracy is referred to as trueness in quantitative classification (Anonymous, 1994). Trueness is defined as the degree of agreement of the expected value with the true value or accepted reference value. This is related to systematic error (Anonymous, 2015a,b). In GMO testing the trueness must be within 25% of the accepted reference value (Anonymous, 2015b). There are no fixed values of trueness that the novel tested qPCR method must meet in microbiological diagnostics. This might be caused by the fact that the trueness in qPCR can be determined by the comparison with some certified reference material, with the reference method or by proficiency testing. Certified reference material with a quantified number of target organisms is available only for a limited number of organisms (especially viruses like HIV, HBV, HCV, HAV, HPV, CMV, EBV), while for the remainder of clinically significant organisms, these materials are often available only for the qualitative analysis, and are therefore not suitable for trueness determination. Reference methods usually have varying diagnostic sensitivities and specificities and often they do not fit for the purposes of the quantitative assessment of novel qPCR methods. Moreover, the organization of proficiency testing via ring trials is expensive and requires a supplier of the reference material (like QCMD). These are the main reasons why determination of trueness in qPCR methods for the microbial detection in clinical, and especially in veterinary food safety areas, is rather limited.

### Precision of qPCR

Precision is defined as the degree of agreement of measurements under specified conditions. The precision is described by statistical methods such as SD or confidence limit (Anonymous, 2015a). From the definition of precision, it is evident that this qPCR parameter is quantitative. For practical determination of precision, two conditions termed repeatability, and reproducibility were introduced (Anonymous, 1994). These two parameters are used to describe the variability of measurements introduced by the operator, equipment, and its calibration, environmental factors that can influence the measurement like temperature, humidity etc., and time between measurements (Anonymous, 1994). Repeatability is described as the closeness of agreement between successive and independent results obtained by the same method on identical test material under the same conditions (apparatus, operator, laboratory, and short intervals of time) and expresses within-laboratory variations (Anonymous, 1994, 2009, 2015a). Repeatability consists of two different variables: intra- and inter-assay variation. The intra-assay variation describes the variability of the replicates conducted in the same experiment; the inter-assay variation describes the variability between different experiments conducted on different days. Numerically, the repeatability is characterized as the SD of replicates at each concentration of each matrix for each method (Anonymous, 2012). The interval characterized by the SD of the replicates is called the repeatability limit (r) and is defined as the value less than or equal to the expected absolute difference, with a probability of 95%, between two tests results obtained under repeatability conditions (Anonymous, 1994, 2009, 2015b; Broeders et al., 2014). If the measured value lies outside the SD, it should be considered as suspect (Anonymous, 2009). It is necessary to perform the estimation of repeatability on 15 repeats at least (Anonymous, 1994, 2015b). Testing of repeatability requires analysis of the spiked relevant matrix at least at four levels—high, medium, low (near to the LOD) and negative in at least duplicates (Anonymous, 2009). For more rigorous testing the use of five replicates and the addition of one more sample spiked with a competitor strain that gives similar results in the given detection system is recommended. Natural background microflora can fulfill this requirement as long as they are present in the matrix at a level 1 Log_10_ greater than the target analyte (Anonymous, 2015a). In clinical, veterinary and food microbial detection, there are no specific recommendations for the repeatability *SD* value in terms of its proportion with respect to the mean. In GMO detection the repeatability *SD* must be ≤25% established on samples containing 0.1% GM related to the mass fraction of GM material (Broeders et al., 2014; Anonymous, 2015b).

On the other hand, reproducibility is the closeness of agreement between single test results on identical test material using the same method, obtained in different laboratories using different equipment and expresses the variation between laboratories (Anonymous, 1994, 2009, 2015a). Numerically, the reproducibility is characterized as the *SD* replicates at each concentration for each matrix across all laboratories (Anonymous, 2012). Similarly to repeatability, the reproducibility limit (R), as the interval characterized by the *SD* of the replicates, is defined as a value less than or equal to which the absolute difference between two test results obtained under reproducibility conditions is expected to have a probability of 95% (Anonymous, 1994, 2009, 2015b). If the difference between two results from different laboratories exceeds R, the results must be considered suspect (Anonymous, 2009). The reproducibility is usually defined by collaborative studies, which determine the variability of the results obtained by the given method in different laboratories using identical samples (Anonymous, 2009, 2012; Molenaar-de Backer et al., 2016). The number of laboratories with valid results which should be included in the collaborative study is at least eight. Therefore, it is advisable to select 10–12 labs (Anonymous, 2009, 2015a). The requirements for the minimal number of testing samples are identical to the repeatability determination (Anonymous, 2009, 2015a). Similarly, there are no specific recommendations for *SD* values of reproducibility with regard to the mean in clinical, veterinary, and food microbial detection. Again, in GMO testing the *SD* of reproducibility should be <35% over the whole dynamic range. However, at relative concentrations <0.2% or at an amount <100 copies *SD* values <50% are deemed acceptable (Broeders et al., 2014; Anonymous, 2015b).

Although determination of qPCR precision requires quantitative data, there is also the possibility of determining the precision of the method qualitatively. The mechanism of precision determination remains identical as for the quantitative estimation, including the validation within collaborative studies. However, the results are evaluated only qualitatively (positive/negative). This approach can be used for the validation of the specific new qPCR method in different laboratories, but it is preferably used for the validation and routine control of various qPCR methods in different laboratories on a set of reference samples. Such samples are provided by certain authorities (reference laboratories) or private companies (QCMD), which collect data from different laboratories and in the case of success, provide certificates regarding participation in such testing.

## Conclusion

qPCR technology represents a powerful tool in microbial diagnostics. In viral and parasitical detection, quantification and typing, the suitability of this technique is beyond doubt; in the area of bacterial diagnostics it can replace culture techniques, especially when rapid and sensitive diagnostic assays are required. The spread of qPCR to different areas of routine microbial diagnostics together with the lack of standard procedures for the determination of basic functional parameters of qPCR has led to a scenario in which standardization of methods is performed according to different rules by different laboratories. This issue was partially solved by the publication of MIQE guidelines (Bustin et al., 2009); however, there are differences in attitude to validation and standardization of qPCR assays across clinical, veterinary and food safety areas. Any contribution to the unification of standardization and validation procedures will improve the quality of qPCR assays in microbial detection, quantification and typing.

## Author contributions

Both authors listed, have made substantial, direct and intellectual contribution to the work, and approved it for publication.

## Funding

The work was supported by the MA CR RO0516 and MEYS CR NPU I program (LO1218). The funder had no role in study design, data collection and interpretation, or the decision to submit the work for publication.

### Conflict of interest statement

The authors declare that the research was conducted in the absence of any commercial or financial relationships that could be construed as a potential conflict of interest.
